# Lipid Nanoparticles Loading Steroidal Alkaloids of Tomatoes Affect Neuroblastoma Cell Viability in an In Vitro Model

**DOI:** 10.3390/pharmaceutics15112573

**Published:** 2023-11-02

**Authors:** Debora Santonocito, Agatina Campisi, Rosalia Pellitteri, Giovanni Sposito, Manuela Giovanna Basilicata, Giovanna Aquino, Giacomo Pepe, Maria Grazia Sarpietro, Maria Gaetana Giovanna Pittalà, Aurelie Schoubben, Rosario Pignatello, Carmelo Puglia

**Affiliations:** 1 Department of Drug and Health Sciences, University of Catania, Viale Andrea Doria 6, 95125 Catania, Italy; campisag@unict.it (A.C.); giovanni.sposito@unict.it (G.S.); mg.sarpietro@unict.it (M.G.S.); rosario.pignatello@unict.it (R.P.); 2 NANOMED-Research Center on Nanomedicine and Pharmaceutical Nanotechnology, University of Catania, 95125 Catania, Italy; 3 Institute for Biomedical Research and Innovation (IRIB), National Research Council, Via P. Gaifami 18, 95126 Catania, Italy; rosalia.pellitteri@cnr.it; 4 Department of Pharmacy, University of Salerno, Via G. Paolo II 132, 84084 Fisciano, SA, Italy; mbasilicata@unisa.it (M.G.B.); gaquino@unisa.it (G.A.); gipepe@unisa.it (G.P.); 5 PhD Program in Drug Discovery and Development, University of Salerno, 84084 Fisciano, SA, Italy; 6 Laboratory of Organic Mass Spectrometry, Department of Chemical Sciences, Viale Andrea Doria 6, 95125 Catania, Italy; mariagaetana.pittala@unict.it; 7 Department of Pharmaceutical Sciences, University of Perugia, 06123 Perugia, Italy; aurelie.schoubben@unipg.it

**Keywords:** tomatine, tomatidine, lipid nanoparticles, cellular viability

## Abstract

Tomato by-products represent a good source of phytochemical compounds with health properties, such as the steroidal glycoalkaloid α-tomatine (α-TM) and its aglycone tomatidine (TD). Both molecules have numerous beneficial properties, such as potential anticancer activity. Unfortunately, their therapeutic application is limited due to stability and bioavailability issues. Therefore, a valid strategy seems to be their encapsulation into Solid Lipid Nanoparticles (SLN). The nanoformulations containing α-TM (α-TM-SLN) and TD (TD-SLN) were prepared by *solvent-diffusion* technique and subsequently characterized in terms of technological parameters (particle size, polydispersity index, zeta potential, microscopy, and calorimetric studies). To assess the effect of α-TM and TD on the percentage of cellular viability in Olfactory Ensheathing Cells (OECs), a peculiar glial cell type of the olfactory system used as normal cells, and in SH-SY5Y, a neuroblastoma cancer cell line, an MTT test was performed. In addition, the effects of empty, α-TM-SLN, and TD-SLN were tested. Our results show that the treatment of OECs with blank-SLN, free α-TM (0.25 µg/mL), and TD (0.50 µg/mL) did not induce any significant change in the percentage of cell viability when compared with the control. In contrast, in SH-SY5Y-treated cells, a significant decrease in the percentage of cell viability when compared with the control was found. In particular, the effect appeared more evident when SH-SY5Y cells were exposed to α-TM-SLN and TD-SLN. No significant effect in blank-SLN-treated SH-SY5T cells was observed. Therefore, SLN is a promising approach for the delivery of α-TM and TD.

## 1. Introduction

In the last century, industrial development and food processing have been responsible for generating great amounts of waste per year; this represents a big problem for the environment [1]. In response to this scenario, the European Union (EU) has promoted the circular economy (CE) theory as a promising strategy for recovering food waste, reducing pollution, and designing more sustainable production chains [2,3]. Therefore, the aim of CE is to give a “second life” to products that are normally considered “waste” and to turn them into a valuable resource.

Currently, there is remarkable attention towards the recovery of by-products of food processing, in particular plant matrices, as possible sources of bioactive compounds with health properties [4]. It has been shown that the main food wastes contain different bioactive molecules, and for this reason, they can be recovered and re-used in various fields. As previously reported, in the agribusiness sector, substantial quantities of waste are generated; a typical example is tomato processing.

**Figure 1:**
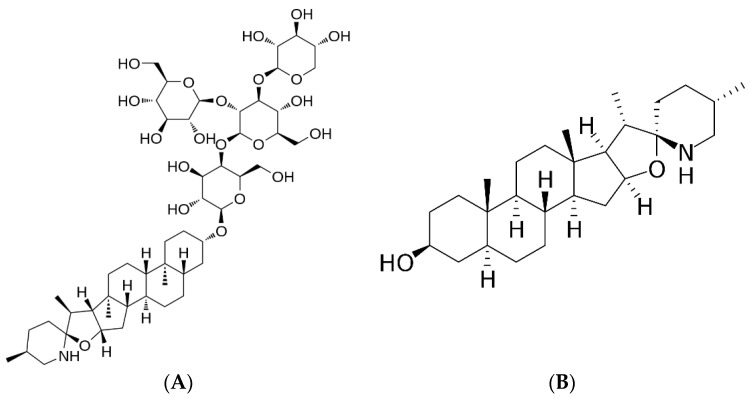
Chemical structure of (**A**) tomatine and (**B**) tomatidine.

The tomato is a plant belonging to the *Solanaceae* family, and it is widely cultivated in the Mediterranean basin. As with many natural compounds [5,6], it is known for its beneficial properties (antioxidant, anti-inflammatory, anti-proliferative, anti-cancer) due to its bioactive constituents. In the agri-food sector, tomatoes are widely processed to obtain juices and purees, thus generating a huge amount of food waste (leaves, peels, and seeds). It has been shown that these tomato by-products, in particular the leaves [7], represent a good source of phytochemical compounds with health properties, such as the steroidal glycoalkaloid α-tomatine (α-TM) and its aglycone tomatidine (TD) (Figure 1). Both bioactive compounds are extracted from the flowers, leaves, and roots of tomato species; moreover, they are localized in the peels of unripe tomatoes [8]. It has been reported that they have numerous beneficial properties, such as antioxidant, antiviral, anti-inflammatory, antibacterial, and anticancer [9,10,11,12]. In particular, the latter has been demonstrated in many cancers, like gastric, breast, and prostate cancer [11], as the tomato glycoalkaloids are able to block the nuclear translocation of the transcription factor NFkB and repress the expression of prosurvival proteins such as Bcl-2, Bcl-xL, and c-IAP1 [13]. Therefore, the recovery and study of by-products of tomato processing could be regarded as an interesting source of bioactive compounds for potential applications in the nutraceutical and pharmaceutical fields.

As reported in the literature [14], plant derivatives have many drawbacks, such as poor solubility and low stability due to the rapid degradation/oxidation process, resulting in little or no therapeutic effect. Therefore, the potential therapeutic application of α-TM and TD is limited [15,16]. A widely used strategy for the delivery of natural compounds is their encapsulation into solid lipid nanoparticles (SLN). SLN are colloidal carriers consisting of a biodegradable solid lipid matrix (Generally Recognized As Safe; GRAS) with an average size in the nanometer range [17]. The encapsulation of natural products into these nanosystems has been shown to have numerous advantages, such as protection of the molecules from degradation and increased stability and solubility, resulting in improved therapeutic effect and specificity [18,19]. This last point is especially important in cancer therapy due to the numerous side effects on healthy tissue. 

Therefore, the aim of this work was to develop an innovative nanoformulation containing α-TM (α-TM-SLN) and TD (TD-SLN) for the potential treatment of cancer. Firstly, α-TM-SLN and TD-SLN were characterized in terms of mean diameter, polydispersity index (PDI), zeta potential (ZP), differential scanning calorimetry (DSC), and long-term stability. Subsequently, an in vitro study was carried out to assess the effect of empty, α-TM-SLN, and TD-SLN on cell viability using OECs, a peculiar glial cell type of the olfactory system used as normal cells, and SH-SY5Y, a neuroblastoma cell line. 

## 2. Materials and Methods

### 2.1. Materials

Tomatine (α-TM) and tomatidine (TD) were purchased from Extrasynthese (Genay, France), soy lecithin (Lecinol S-10) was obtained from Nikko Chemical (Milan, Italy), and poloxamer 188 (Lutrol F68) was provided by BASF Chem-Trade GmbH (Burgbernheim, Germany). Mice pups were provided by Envigo RMS s.r.l., Italy, stock: C57BL6. SH-SY5Y The human neuroblastoma cell line was from the Cell Bank Interlab Cell Line Collection (ICLC) (Genova, Italy). Antibiotics, Trypsin, non-Essential Amino Acids, Phosphate Buffer Saline solution (PBS), cytosine arabinoside, health-inactivated Fetal Bovine Serum (GIBCO), and Modified Eagle Medium (MEM) with 2 mM GlutaMAX were from GIBCO (Milan, Italy). Stearic acid, hydroxypropylmethyl cellulose (HPMC K15M), Ham’s F12, [3(4,5-dimethyl-thiazol-2-yl)2,5-diphenyl-tetrazolium bromide), MTT] and other chemicals were purchased from Merck (Milan, Italy). All solvents and LCMS-grade additives were purchased from VWR Chemicals (Milan, Italy).

### 2.2. α-TM-SLN and TD-SLN Preparation

α-TM-SLN was prepared by the *solvent-diffusion* technique, a method widely used for the delivery of natural compounds [20,21]. Briefly, HPMC K15M (230 mg), Lecinol S-10 (230 mg), and Lutrol F68 (230 mg) were dissolved in hot deionized water (70 °C). The melted lipid phase (70 °C), composed of lipid matrix (stearic acid 23 mg), α-TM (7.08 mg), and ethanol (4.6 mL), was dropped in the hot aqueous phase by using a high-speed stirrer (Ultra-Turrax T25, IKA-Werke GmbH &Co. Kg, Staufen, Germany) at 15,000 rpm for 8 min. Subsequently, an ultrasonic system (UP 400 S, Dr. Hielscher GmbH, Stuttgart, Germany) was used for 10 min to form a nanoemulsion. Finally, the obtained nanoemulsion was cooled in an ice bath for 5 min, and the ethanol was evaporated. TD-SLN were formulated with the same procedure by replacing α-TM with TD (1.4 mg), while empty SLN were formulated with the same procedure without the addition of α-TM or TD.

### 2.3. α-TM-SLN and TD-SLN Characterization

A Zetasizer Nano-ZS90 (Malvern Instrument Ltd., Worcs, UK) was used to measure the mean particle size (Z-average), polydispersity index (PDI), and zeta potential (ZP) of the samples at 20 °C. All formulations were diluted 10-fold with deionized water for measurement. All samples were measured in triplicate. Moreover, these nanotechnological parameters (mean particle size, PDI, and ZP values) were monitored at specific time points (0, 7, 14, 21, 30, 60, 90, 120, 150, and 180 days) for 180 days after storage of all formulations at room temperature.

### 2.4. α-TM-SLN and TD-SLN Morphology

Lipid nanoparticle morphology was investigated using Scanning Electron Microscopy (SEM) with a Field Emission SEM (LEO 1525 equipped with a GEMINI column, ZEISS, Germany). Samples were prepared by dropping the diluted nanoparticle suspension on a circular microscope slide that was first deposited onto an aluminum specimen stub covered with a double-sided adhesive carbon disc. The samples were sputter coated with chromium before imaging (100 mA, 24 s, 8 nm thickness) (Quorum Q150T ES East Grinstead, West Sussex, UK). Nanoparticle morphology was further confirmed by Transmission Electron Microscopy (TEM) (Philips EM 400 T microscope, Eindhoven, Nederland). Samples were prepared by laying a drop of the diluted nanoparticle suspension onto a 200-mesh Formvar^®^ coated copper grid (TAAB Laboratories Equipment Ltd., Aldermaston, UK). Samples were stained using 0.5% phosphotungtic acid to improve contrast and allow particle observation. The samples were then allowed to dry overnight at 20 °C before analysis.

### 2.5. Differential Scanning Calorimetry (DSC)

The calorimetric analysis was performed using a Mettler Toledo STAR^e^ system (Switzerland) equipped with a DSC^1^ calorimetric cell. Mettler TA-STAR^e^ software (version 16.00) was used to obtain and analyze the data. The sensitivity was automatically chosen as the maximum possible by the calorimetric system. The calorimeter was calibrated using 99.95% indium. A total of 120 µL of samples were transferred to hermetically sealed 160 µL DSC aluminum crucibles and subjected to calorimetric analysis under nitrogen flow (70 mL/min) as follows:

- Heating from 25 °C to 85 °C, heating rate: 2 °C/min.
- Cooling from 85 °C to 25 °C, cooling rate: 4 °C/min.

All samples were measured in triplicate to verify the reproducibility of the results.

### 2.6. In Vitro Release Study 

A release study was performed using Franz diffusion cells (LGA, Berkeley, CA, USA) with a cellulose acetate membrane (0.2 µm pore size, 25 mm diameter, Sartorius; Göttingen, Germany). The receptor phase, composed of an ethanol-water mixture (60:40 *v*/*v*), since α-TM and TD have low water solubility, was thermostated at 35 °C under stirring. 1 mL of each formulation was applied to the membrane surface, and the experiments were run in duplicate for 24 h. At intervals (0, 2, 4, 6, 8, 22, and 24 h), 200 µL of receptor phase were taken and replaced with the same volume of fresh solution. The samples of the receptor phase were analyzed using a Shimadzu Nexera UHPLC (Kyoto, Japan) coupled online to a triple quadrupole LCMS-8050 (Shimadzu, Milan, Italy) equipped with an Electrospray Ionization (ESI) source operating in positive mode. The samples were separated on a reversed-phase analytical column (Kinetex^®^ 2.6µm EVO C18 100 Å, LC Column 50 × 2.1 mm, Phenomenex, Bologna, Italy) that was thermostated at 40 °C. The mobile phases consisted of (A) H_2_O and (B) ACN, both acidified with 0.1% *v*/*v* HCOOH, delivered at a constant flow rate of 0.4 mL min^−1^. Analyses were performed in gradient elution as follows: 0.0–5.0 min, 5–95% B; 5.00–6.00 min isocratic to 95% B; 6.00–6.01 min returning to 5% B and remained unchanged to the end of the run. The total run time was 9.01 min. 

α-TM and TD quantifications were carried out in MRM (Multiple Reaction Monitoring) mode, monitoring the transition from the protonated precursor to the product. To fine-tune the LC-MS/MS conditions, a standard solution with a concentration of 1 µg mL^−1^ in MeOH was introduced into the mass spectrometer using the flow injection mode. The transitions of α-TM (Figure S1) were *m*/*z* 1034.2 > 255.25 (quantifier ion), *m*/*z* 1034.2 > 416.3, and *m*/*z* 1034.2 > 578.3 (qualifier ions). The transitions of TD (Figure S1) were *m*/*z* 416.1 > 161.25 (quantifier ion), *m*/*z* 416.1 > 398.3, and *m*/*z* 416.1 > 273.25 (qualifier ions). The dwell time was set to 50 ms for all the monitored transitions. Interface temperature, DL temperature, and Heat Block temperature were set to 300 °C, 250 °C, and 350 °C, respectively. Nebulizing gas, heating gas, and drying gas flow were set to 3, 10, and 10 L min^−1^, respectively. All the data were collected in centroid mode and acquired and processed using Lab Solution workstation software.

### 2.7. Calibration Curve

For the preparation of α-TM and TD calibration curves, stock solutions were diluted with methanol. Working standard solutions were prepared by serial dilution of the stock solution to obtain the required concentrations (1–200 ng mL^−1^). Triplicate injections were performed for each concentration level. Peak areas of the standards were plotted against corresponding concentrations. Linear regression was employed to generate the calibration curve, with R^2^ values ≥ 0.9994 for α-TM and ≥0.9995 for TD. Limits of detection (LOD) and quantification (LOQ) were determined using the standard deviation (SD) and the slope of the calibration curves, multiplied by 3.3 and 10 folds, respectively. The chromatographic system′s repeatability was evaluated in terms of intra-day and inter-day precision. Accuracy was assessed by the percentage relative error (Er %), while precision was evaluated by the percentage of the relative standard deviation (% RSD). The obtained data demonstrated acceptable accuracy and precision for the developed analytical method (Table S1). 

### 2.8. Encapsulation Efficiency (EE%)

#### 2.8.1. Mass Spectrometric Analysis for Quantification of α-TM and TD in SLN Formulations

**Figure 2:**
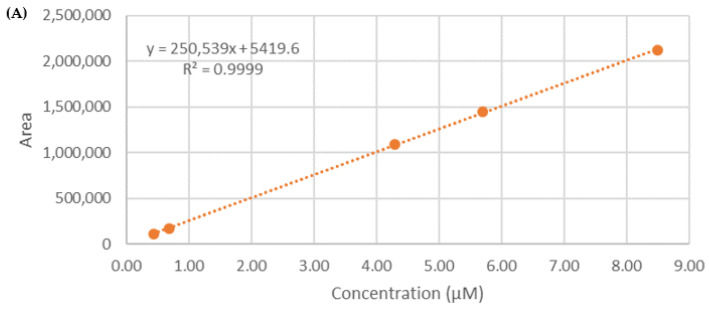

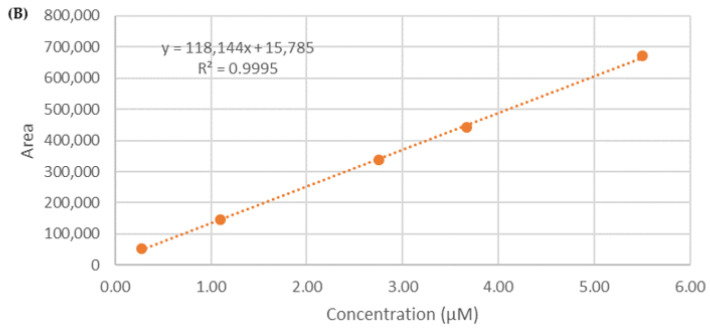
Calibration curves for the quantification of (**A**) α-TM and (**B**) TD in SLNs.

The RP-HPLC/ESI-MS/MS was performed using a Thermo Scientific Dionex UltiMate 3000 RSLC system coupled online with a linear ion trap electrospray mass spectrometer (LTQ, Thermo Finnigan, Milan, Italy). A total of 20 µL of each sample was directly loaded onto a C18 capillary column (Hypersil GOLDTM, 3.0 mm, 1 mm × 100 mm, 3 µm particle size, Thermo Scientific), eluted at room temperature with a linear gradient of solvent B (CH_3_CN + 1% FA) in A (H_2_O + 1% FA) from 5% to 30% in 15 min, followed by 30% to 95% in 5 min and 95% to 5% in 15 min at a flow rate of 50 µL/min. Eluted samples were ionized by an electrospray ion source (in positive polarity) using a spray voltage of 1.9 KV and introduced into the mass spectrometer through a heated ion transfer tube (220 °C). The ions of each sample were analyzed using the data-dependent method as follows: (1) full MS scan (mass-to-charge ratio 151–2000); (2) zoom scan of the three most intense ions (isolation width: 2 Da); and (3) MS/MS analysis of the three most intense ions (Q 0.250, collision energy 35 a.u). Mass spectrometer calibration was performed using the Pierce^®^LTQ Velos ESI Positive Ion Calibration Solution (Thermo Fisher Scientific). MS data acquisition was performed using Xcalibur v. 3.0.63 (Thermo Fisher Scientific). The quantification of α-TM and TD occurred employing a calibration curve of the standard of the two molecules in the range 0.4–9.0 µM for TT (r^2^ = 0.9999) and 0.2–6.0 µM for TD (r^2^ = 0.9995) (Figure 2). 

#### 2.8.2. Determination of Entrapment Efficiency (EE)

The entrapment efficiency of α-TM and TD into SLN was determined after column chromatography according to a procedure previously developed [22,23]. Briefly, 0.5 mL of each formulation was loaded onto a Sephadex LH20 eluting with: H_2_O (15 mL), H_2_O: EtOH 50:50 (10 mL), EtOH (10 mL), and acetone (10 mL). The EE was determined according to equation 1 after quantification of α-TM or TD by HPLC/ESI-MS/MS in the evaluation of the first fraction.

EE% = (µM_det_ ÷ µM_tot_) × 100 (1)

µM_det_ is the concentration (µM) determined by HPLC/ESI-MS/MS, and µM_tot_ is the concentration employed for the preparation of SLN. The procedure was repeated three times, and the data were expressed as the mean (*n* = 3) ± SD.

### 2.9. In Vitro Study on α-TM-SLN and TD-SLN

#### 2.9.1. Primary OEC Cultures

Olfactory bulbs were derived from mouse pups (P2) and processed according to the method described by Pellitteri et al. [24,25]. Collagenase and trypsin were used to digest the tissue. Subsequently, DMEM was added with 10% FBS to block trypsinization. Cell suspension was then placed in 75 cm^2^ flasks and fed with complete DMEM. After 24 h, cytosine arabinoside (10-5 M), an antimitotic agent used to reduce the number of dividing fibroblasts, was added. In addition, cell cultures were further purified using the method of Chuah and Teague [26]. Finally, the cell cultures were incubated at 37 °C in an environment with humidified air and CO_2_ (95-5%). Medium cultures were changed 2–3 times per week. 

#### 2.9.2. SH-SY5Y Cell Line Cultures

SH-SY5Y cell line cultures were obtained through cell suspension in complete culture medium containing: Ham′s F12 and MEM (1:1), 10% (*v*/*v*) FBS, 2 mM GlutaMAX, and 50 mg/mL penicillin/streptomycin (50 U/mL). The cell suspension was plated in 75 cm^2^ flasks at a final density of 2 × 10^6^ cells and incubated at 37 °C in humidified air and CO_2_ (95-5%). The culture medium was changed every 2–3 days. When the cell cultures reached approximately 80–85% confluence, they were subcultured at a density ratio of 1:4 and incubated at 37 °C in a humidified atmosphere containing CO_2_ (95-5%).

#### 2.9.3. Treatment of Cells

Primary OEC and SH-SY5Y cell cultures were exposed for 24 h (optimal exposure time) to the following treatments: A group of cell cultures was treated with α-TM or TD at different concentrations (0.25 µg/mL and 0.50 µg/mL); another group was treated with blank SLNs (0.25 µg/mL); another group was treated with blank SLNs (0.50 µg/mL); other groups were treated with α-TM loaded into SLNs (0.25 µg/mL) or TD loaded into SLNs (0.50 µg/mL); a group was treated with a corresponding volume of PBS (final concentration 0.01% *v*/*v*), used as a control.

#### 2.9.4. MTT Assay

To monitor cell viability, the MTT test was used [27,28,29,30]. Briefly, cells were set up at 0.5 × 10^4^ cells per well of a 96-multiwell, flat-bottomed, 200-µL microplate and maintained at 37 °C in a humidified air mixture and CO_2_ (95-5%). At the end of treatment time, 20 µL of 0.5% MTT in (pH 7.4) PBS was added to each microwell. After 2 h, the supernatant was removed and replaced with 100 µL of DMSO. The optical density of each one was measured with a microplate spectrophotometer reader (Titertek Multiskan; Flow Laboratories, Helsinki, Finland) at λ = 570 nm. Data were expressed as a percentage of PBS (control), as taken as 100%, to normalize the values.

### 2.10. Statistical Analysis

To assess the significant differences among groups, data were analyzed through one-way analysis of variance (ANOVA) followed by a post hoc Holm–Sidak. Results were reported as the mean ± SD of four separated experiments performed in triplicate, and differences between groups were considered to be significant at * *p* < 0.05.

## 3. Results

### 3.1. Characterization of α-TM-SLN and TD-SLN

α-TM-SLN and TD-SLN were formulated by the *solvent-diffusion* technique, using stearic acid as the lipid matrix [20]. The nanotechnological parameters (mean particle size, polydispersity index, and zeta potential) were measured by Dynamic Light Scattering (DLS) analysis. As reported in Table 1, all nanoformulations showed an average particle size of about 125 nm, a polydispersity index (PDI) of around 0.24, and a zeta potential (ZP) of about −25 mV. Empty SLN showed a lower size (121.2 ± 0.31) but a larger PDI (0.261 ± 0.02) [31].

**Figure 3:**
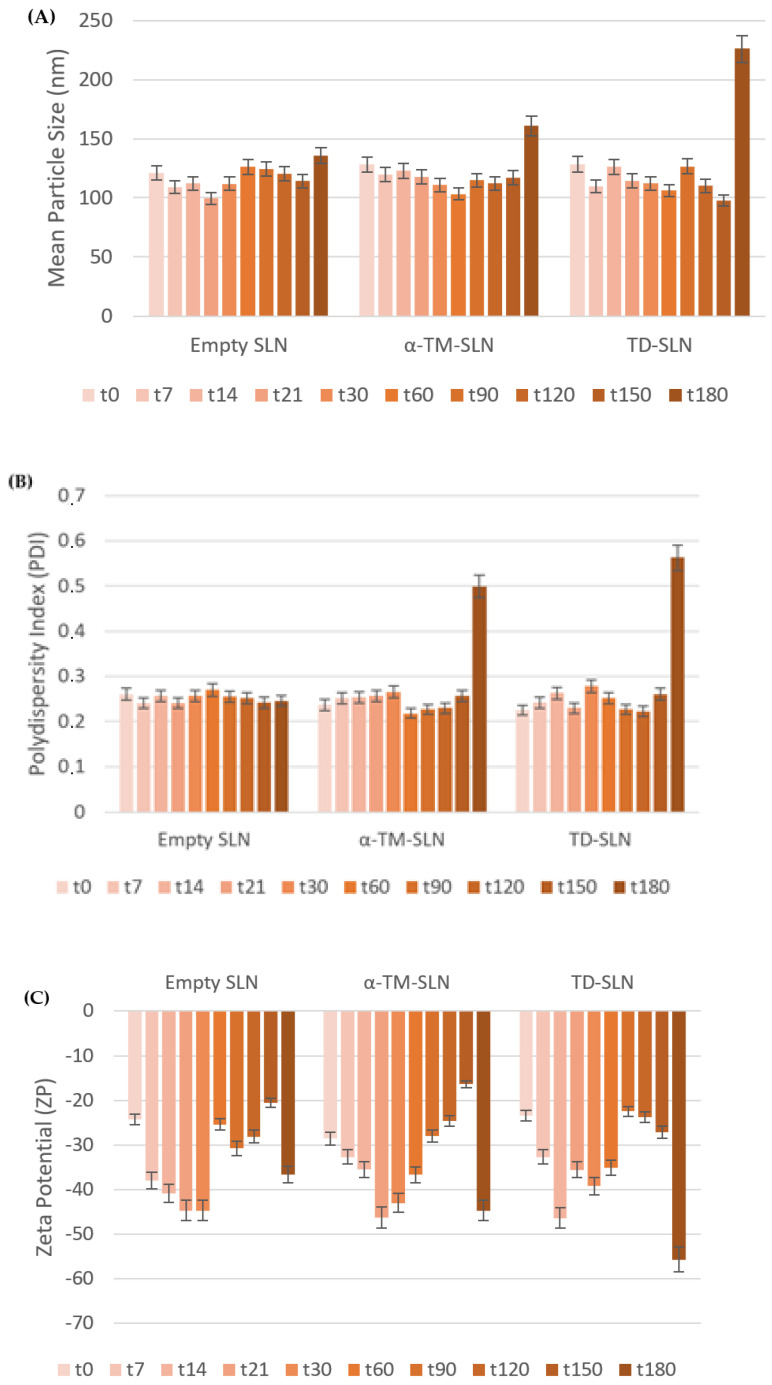
(**A**) Mean particle size; (**B**) polydispersity index (PDI); and (**C**) Z-potential of empty, α-TM-SLN, and TD-SLN during storage at room temperature for 180 days.

The ZP value predicted the good storage physical stability of the formulation; this data were further confirmed by long-term stability studies (180 days) [32]. In fact, these findings showed that all formulations had acceptable long-term stability until 150 days of storage at room temperature (Figure 3). The same results were obtained during storage at +4°.

### 3.2. Morphology of α-TM-SLN and TD-SLN

**Figure 4:**
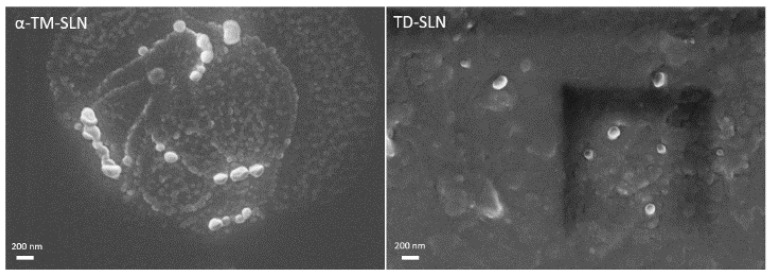
Scanning electron microscopy images of α-TM-SLN and TD-SLN. The scale bar represents 200 nm.

**Figure 5:**
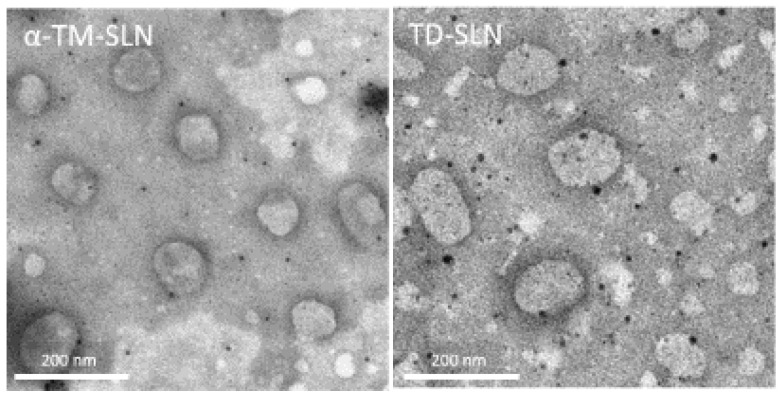
Transmission electron microscopy images of α-TM-SLN and TD-SLN. The scale bar represents 200 nm.

SEM photomicrographs of lipid nanoparticles show particles characterized by an almost spherical shape and a smooth surface (Figure 4). Both α-TM-SLN and TD-SLN observed using SEM have dimensions compatible with those determined using photon correlation spectroscopy. Figure 5 shows TEM photomicrographs of SLN. Also, with this microscopy technique, SLN appears to have a shape close to sphericity, and its sizes are in agreement with the particle dimension analysis performed. Both microscopy techniques allow for the confirmation of the SLN features determined using the Zetasizer Nano-ZS90.

### 3.3. Differential Scanning Calorimetry (DSC)

**Figure 6:**
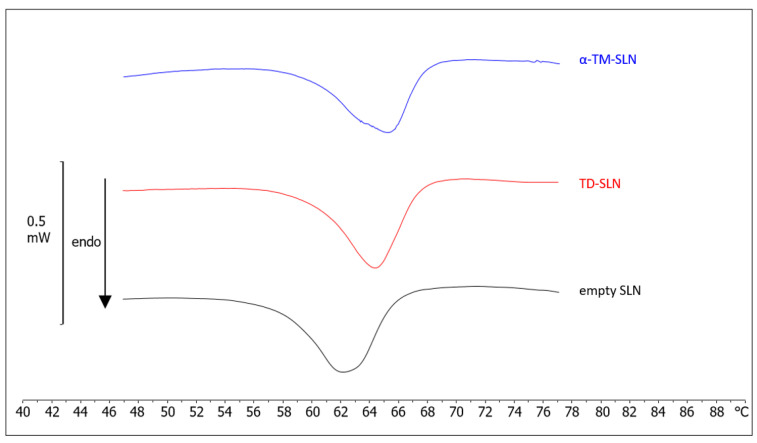
Calorimetric curves, in heating mode, of empty SLN, α-TM-SLN, and TD-SLN.

Unloaded SLN, α-TM-SLN, and TD-SLN were subjected to calorimetric analysis following the protocol described at 2.5 (Figure 6). Unloaded SLN showed a broad, yet well-defined thermogram, with a peak temperature of about 62 °C and an enthalpy variation of −29 J/g. α-TM-SLN had an enthalpy variation of −25 J/g, a shift of peak at a higher temperature (about 65.5 °C), and the appearance of a shoulder at 63 °C. The results demonstrated that α-TM interacted with SLN, possibly due to its encapsulation in the SLN structure. In particular, the presence of the shoulder suggested a phase separation, which could be due to α-TM-rich and poor regions; furthermore, the main peak at higher temperatures suggested a stabilization of SLN structure due to the presence of α-TM. Instead, TD-SLN exhibited a unique peak at 64.36 °C with an enthalpy variation of −27.00 J/g, which is evidence of the encapsulation and a uniform distribution of the compound in the SLN. 

### 3.4. Determination of Entrapment Efficiency (EE%)

The EE% of the SLN was determined by HPLC/ESI-MS/MS, applying a procedure previously reported based on gel filtration chromatography on Sephadex-LH20 [22,23]. In the conditions reported in the experimental section, α-TM-SLN and TD-SLN were eluted in the aqueous fraction, thus allowing the quantification of entrapped molecules in SLNs. The entrapment efficiency was calculated directly according to equation (1). The results achieved indicated good entrapment efficiency, as the EE% of α-TM and TD in SLN was 72.1% ± 3.6 and 67.8% ± 2.3, respectively.

### 3.5. In Vitro Release Study

**Figure 7:**
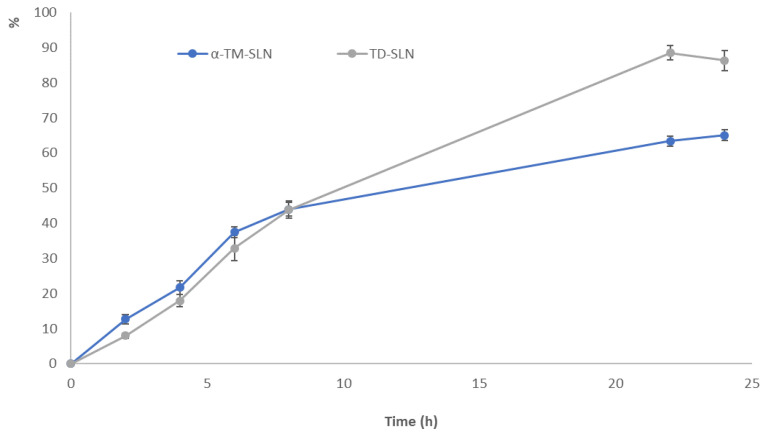
α-TM and TD-loaded SLN release samples.

The release profiles of α-TM and TD from the SLN formulations were measured in vitro over 24 h. As shown in Figure 7, α-TM-SLN and TD-SLN exhibited a slow-release property, which is an important feature for a product with long-lasting function. This is due to the slow release of molecules that are successfully encapsulated within the lipid core. The results showed that the maximum α-TM (126.4 ng/mL) and TD (31.62 ng/mL) amounts, corresponding to approximately 65% and 88% of the loaded drug, were reached after 22 h, followed by a slower release until the end of the experiment [33].

### 3.6. Percentage of Cell Viability of OEC and SH-SY5Y Cultures

**Figure 8:**
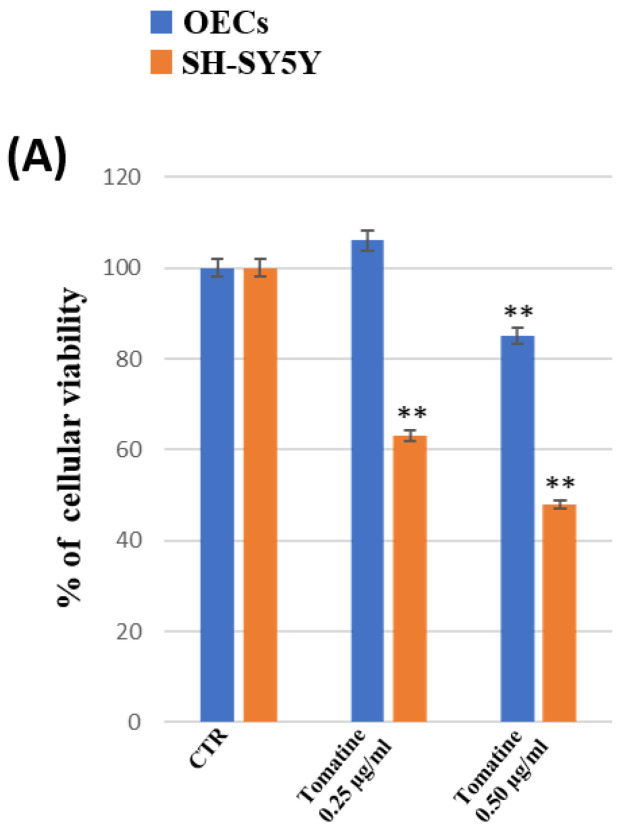

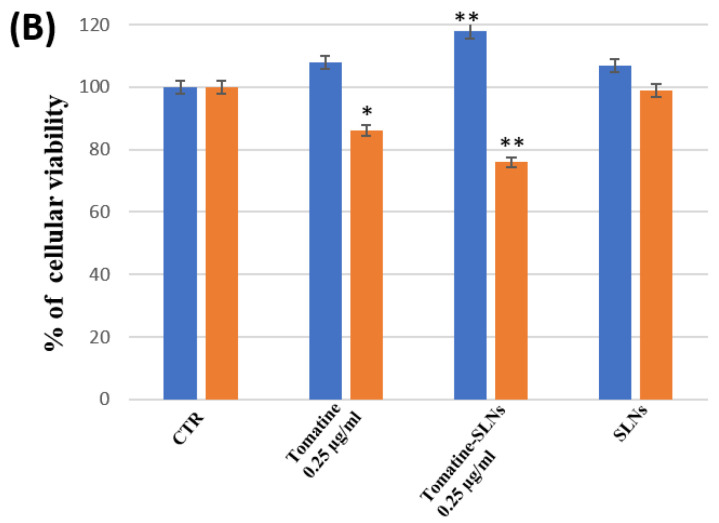
MTT tests were performed on OECs (blue) and SH-SY5Y (orange). (**A**) Untreated cell (CTR), α-TM at 0.25 µg/mL and 0.50 µg/mL for 24 h. (**B**) OECs and SH-SY5Y were treated with blank SLN, α-TM 0.25 µg/mL, and α-TM-SLN 0.25 µg/mL for 24 h. * *p* < 0.05 difference vs. CTR; ** *p* < 0.001 vs. CTR.

**Figure 9:**
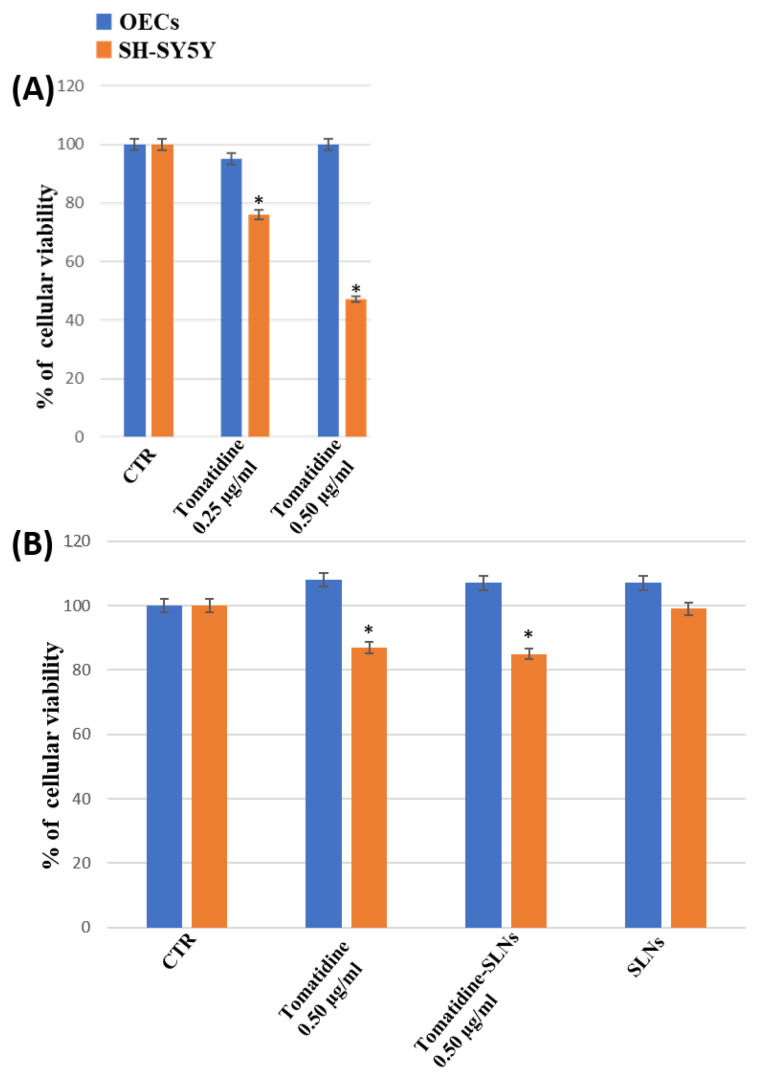
MTT tests were performed on OECs (blue) and SH-SY5Y (orange). (**A**) Untreated cell (CTR), TD at 0.25 µg/mL and 0.50 µg/mL for 24 h. (**B**) OECs and SH-SY5Y were treated with blank SLN, TD 0.25 µg/mL, and TD-SLN 0.25 µg/mL for 24 h. * *p* < 0.05 difference vs. CTR.

To monitor the percentage of cell viability on OECs, a glial cell type of the olfactory system used as normal control cells, and SH-SY5Y cells, a neuroblastoma cancer cell line, both in the absence and in the presence of free α-TM or TD at different concentrations (0.25 µg/mL; 0.50 µg/mL) and once encapsulated into SLNs for 24 h (optimal experimental condition, as in longer times the cells were very confluent), an MTT test was performed. Figure 8A shows that the treatment of OECs for 24 h with free α-TM at a concentration of 0.25 µg/mL did not induce any significant change in the percentage of cell viability when compared with the control. In contrast, a significant decrease in the percentage of cell viability was observed when the cells were treated with 0.50 µg/mL of α-TM. As shown in Figure 9A, OECs-treatment with 0.25 µg/mL or 0.50 µg/mL TD did not cause any significant modification of the percentage of cell viability when compared with the control. Thus, we chose to expose OECs to 0.25 µg/mL of α-TM and 0.50 µg/mL of TD for 24 h. When the cells were treated with 0.25 µg/mL α-TM-SLN, a significant increase in the percentage of cell viability was found when compared with the control (Figure 8B). Otherwise, no significant change between untreated OECs and 0.50 µg/mL TD-SLN was observed (Figure 9B). In contrast, in the neuroblastoma cancer cell line SH-SY5Y, treatment with 0.25 µg/mL or 0.50 µg/mL of free α-TM induced a significant decrease in the percentage of cell viability when compared with the control (Figure 8A). The effect appeared more evident when cell cultures were exposed to 0.25 µg/mL of α-TM-SLN. The treatment with 0.50 µg/mL TD was able to significantly reduce the percentage of cell viability when compared with the controls (Figure 9). When SH-SY5Y cell cultures were treated with 0.50 µg/mL TD-SLN, a very slight decrease in the percentage of cell viability when compared with free TD was found (Figure 9B). Both OECs and SH-SY5Y cell cultures treated with empty SLN did not show any change in the percentage of cell viability.

## 4. Discussion

Although glycoalkaloids are generally considered toxic, α-tomatine (α-TM) and tomatidine (TD) appear to be well tolerated in humans. In fact, they have many beneficial properties, such as the ability to inhibit cancer cell growth in in vitro studies [34,35].

Since there are no articles in the literature concerning the delivery of α-TM and TD using lipid nanoparticles, it was decided to use a method widely studied by our research group [20,21]. α-TM-SLN and TD-SLN were formulated by the *solvent-diffusion* technique, a method that has been proven to be suitable for the encapsulation of both molecules and highly reproducible. Stearic acid was selected as the lipid matrix since it was shown in our previous work that it did not cause cytotoxic effects on cells [20,36]. As reported in Table 1, all nanoformulations showed a small particle size of about 125 nm, a polydispersity index (PDI) of around 0.24, and a zeta potential (ZP) of about −25 mV. The latter value suggests acceptable long-term stability [31,37], subsequently confirmed by monitoring these parameters over time at room temperature (Figure 2). Although SLN generally showed some stability issues (particle size enhancement, drug expulsion from the lipid matrix) [38], all formulations did not show significant changes in mean size and PDI until 150 days of storage. Regarding ZP, significant variations were observed, probably due to the release of encapsulated compounds and/or the structural rearrangement of lipid colloidal systems under storage conditions. Furthermore, the transparent appearance of lipid formulations was maintained during 150 days of storage. 

The entrapment efficiency (EE%) was calculated indirectly according to Equation (1). This method was previously studied by our research group, proving to be valid and highly reproducible for both molecules. The achieved results agree with our previous article, which indicated an entrapment efficiency of around 70% [22].

In terms of drug release, our results are in accordance with a published study on saquinavir-loaded SLN [39]. As revealed in Figure 7, α-TM and TD were released gradually from SLN. In the initial phase, the amounts of released drugs were entirely insignificant, as the majority of α-TM and TD were distributed in lipid cores. Subsequently, sustained drug release was achieved; this indicated that a lipid core is able to decelerate the delivery of drugs and obtain a formulation with a slow-release property.

Differential scanning calorimetry (DSC) is a valuable technique to study the thermotropic behavior of lipid nanoparticles. It measures the heat exchanges associated with structural alterations of materials and allows for the acquisition of information on the structural properties of the samples [40]. In this study, DSC was used to characterize unloaded SLN, α-TM-SLN, and TD-SLN. Both α-TM and TD affected the thermotropic behavior of SLN, suggesting their insertion in the SLN structure. However, the calorimetric profiles of α-TM-SLN and TD-SLN are different, which indicates a different localization of the molecules in the SLN. α-TM-SLN showed two signals (one shoulder and one peak) on a curve indicative of a not uniform distribution of α-TM in the SLN. Taking into account the molecule structure, it can be hypothesized that it mainly localizes next to the interface lipid/surfactant, with the glyconic moiety towards the surfactant molecules; in this way, the lipid core will be poor in α-TM whereas the lipid shell will be rich in α-TM. TD-SLN shows a unique signal, which indicates that TD is distributed homogeneously among the lipid molecules. 

It is known that there is a correlation between a diet rich in tomatoes and the risk of cancer. This effect is due to the lycopene content, a strong natural antioxidant. [8,41]. Several in vivo and in vitro studies have reported the antiproliferative activity of α-TM and TD against cancer cell lines [10,42]. In this work, we investigated the effect of empty, α-TM-SLN, and TD-SLN on cell viability using OECs, a peculiar glial cell type of the olfactory system, as normal cells, and SH-SY5Y, a neuroblastoma cell line.

We chose Olfactory Ensheathing Cells (OECs) as a control because they possess proliferative activity and stem cell characteristics and represent a source of multiple trophic factors that play a decisive role in Central Nervous System (CNS) regeneration [43]. Furthermore, OECs are particular glial cells of the olfactory nerve that are involved in the uptake of intranasal administration. In the last decades, the nasal route has deserved great attention as a convenient and safe route for the CNS target in the administration of drugs [44]. Moreover, we selected SH-SY5Y neuroblastoma cells to observe the neuroprotective effect of α-TM and TD, as both molecules have shown inhibitory capacity on cholinesterases and are able to interact with nicotinic receptors, in particular type 7 [45]. Therefore, our future perspectives are to study the effectiveness of α-TM-SLN and TD-SLN on the CNS through nose-to-brain delivery. Our results highlighted that both free α-TM and TD treatment did not induce a significant decrease in the percentage of OEC viability when compared with the control. In contrast, when SH-SY5Y cells were treated with α-TM and TD-free or encapsulated in SLN, a significant decrease was observed, inducing us to consider SLN as a promising tool for the delivery of α-TM and TD. Our results, together with others [46], demonstrate that SLN represents a promising tool as a carrier of natural compounds, such as α-TM and TD, protecting these compounds from external factors, improving their stability and bioavailability, and increasing their cell uptake and anticancer activity; the latter is probably due to the block of the nuclear translocation of the transcription factor NFkB [13]. Moreover, it is interesting to highlight the remarkable biocompatibility and biodegradability of lipids used for SLN production, which are able to increase and preserve the benefits of encapsulated compounds. This is less noticeable with the use of other drug delivery systems; in fact, Nepal and coworkers measured the cell viability of α-TM, free and loaded into mesoporous silica nanoparticles (MSN), on liver cancer cells, demonstrating a comparable level of toxicity between them [47]. This indicated that the use of MSN did not increase the anticancer activity of α-TM.

Therefore, SLN seems to be suitable for the delivery of natural compounds in several applications, including cosmetic and pharmacologic fields.

Further in vitro and in vivo studies will be conducted to observe the molecular biological mechanisms of both molecules, free and loaded into SLN, in order to highlight the importance of encapsulation, such as increased bioavailability and crossing of the blood-brain barrier. Moreover, the same studies will be planned on tomato leaf extracts to promote the circular economy.

### Limitation Section

This study has been conducted using standard compounds obtained by a dealer company. We had no indications on the effect of the inclusion of the bioactive compounds from the natural source. We will probably have to consider the effect of the phytocomplex and the potential synergistic effect of all the compounds contained in the extract. Obviously, to obtain further information, the implementation of in vitro evidence or in vivo experiments is necessary. Although this last strategy is not a simple task for economic and ethical issues.

Another point that should be evaluated is related to the formulation and its stability. In order to increase the formulations’ stability, further experiments are underway to study the effect of freeze-drying on the storage time and the stability of the encapsulated compound.

Moreover, we will investigate the effect of resuspension, trying to find the best conditions in order to obtain nanotechnological parameters similar to the pre-lyophilization conditions. With this aim, different cryoprotectants will be screened to select the best storage conditions for our nanosystems.

## 5. Conclusions

α-tomatine (α-TM) and tomatidine (TD), two interesting steroidal alkaloids contained in tomato by-products, have a wide range of beneficial properties, such as anticancer activity. As with many natural compounds, their therapeutic application is limited; therefore, a valid strategy seems to be their encapsulation into Solid Lipid Nanoparticles (SLN). The nanoformulations containing α-TM (α-TM-SLN) and TD (TD-SLN) were prepared by *solvent-diffusion* technique, obtaining spherical nanoparticles with good nanotechnological parameters suitable for all administration routes (<150 nm); in particular, nanoparticles showed good stability for 180 days. Furthermore, DSC studies were performed to study the interactions between the lipid matrix and bioactive compounds, which are evidence of the encapsulation and distribution of the compounds in the SLN. This was further demonstrated by an in vitro release profile showing that both nanoformulations exhibited a slow-release property due to the slow release of molecules that were successfully encapsulated within the lipid core. Biological results demonstrated that the treatment with free α-TM (0.25 µg/mL) and TD (0.50 µg/mL) on SH-SY5Y cultures induced a significant decrease in the percentage of cell viability when compared with the control. In particular, the effect appeared more evident when SH-SY5Y cells were exposed to treatment with α-TM-SLN and TD-SLN, confirming their potential anticancer activity. Therefore, the use of nanotechnology could be regarded as a promising strategy for delivering α-TM and TD and exploiting their potential anticancer properties.

## Figures and Tables

**Table 1:** Mean particle size (Z-Average), polydispersity index (PDI), and zeta potential (ZP) of empty, α-TM-SLN, and TD-SLN.

Sample	Z-Average (nm)	PDI	ZP (mV)
Empty SLN	121.2 ± 0.31	0.261 ± 0.02	−24.3 ± 0.4
α-TM-SLN	128.2 ± 0.26	0.237 ± 0.12	−28.6 ± 0.9
TD-SLN	128.4 ± 0.7	0.225 ± 0.44	−23.5 ± 0.6

## Data Availability

The data presented in this study are available in the article.

## Supplementary Materials

The following supporting information can be downloaded at: https://www.mdpi.com/article/10.3390/pharmaceutics15112573/s1, Figure S1: MRM transition of α-Tomatine (up) and Tomatidine (down); Table S1: Method validation parameters for quantitation of α-TM and TD.
