# Supplementary material for: Lipid Nanoparticles Loading Steroidal Alkaloids of Tomatoes Affect Neuroblastoma Cell Viability in an In Vitro Model
Source: Pharmaceutics. 2023 Nov 2;15(11):2573. doi: 10.3390/pharmaceutics15112573 (PMC10675799; doi:10.3390/pharmaceutics15112573)
Supplement: Supplementary file 1 [file pharmaceutics-15-02573-s001.zip › pharmaceutics-2629175-supplementary.pdf]

Supporting Informations

Formulation and characterization of lipid nanocarriers encapsulating steroidal alkaloids of tomato and evaluation of potential anticancer activity in an in vitro model

Contents:

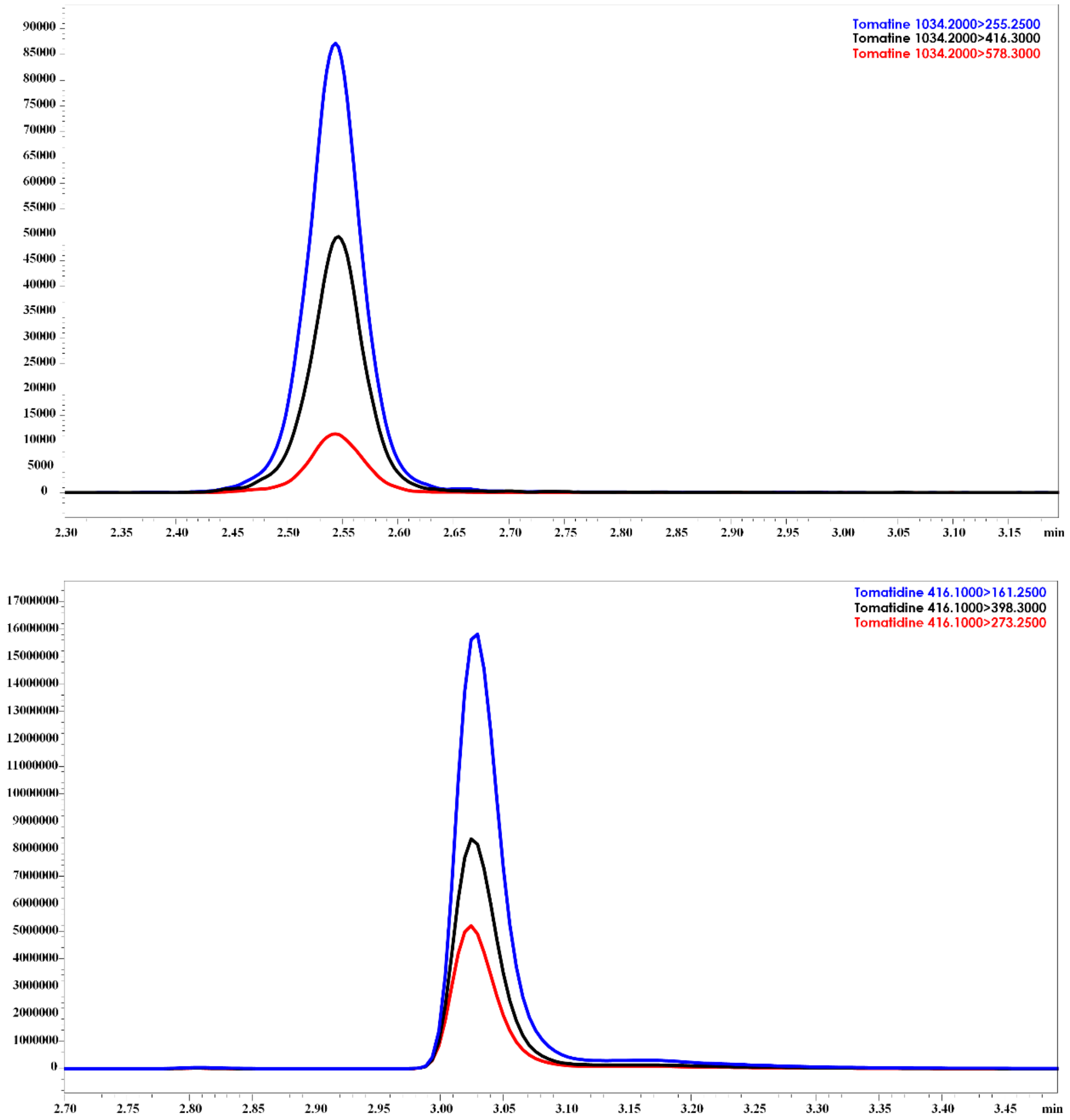

Figure SX1. MRM transition of α-Tomatine (up) and Tomatidine (down).

Table S1. Method validation parameters for quantitation of α-TM and TD.

Parameters	Tomatine		Tomatidine	
**Intercept**	12.794		0.1429	
**Slope**	7.0E-04		1.10E-06	
**Correlation coefficient (R^2)**	≥ 0.9994		≥ 0.9995	
**LOD (ng mL^{-1})**	2.53		0.11	
**LOQ (ng mL^{-1})**	0.83		0.36	
**Intraday (n = 3)**	**Precision (RSD%)**	**Accuracy (Er%)**	**Precision (RSD%)**	**Accuracy (Er%)**
**LQC**	2.7	94.3	2.1	102.7
**MQC**	0.3	93.5	0.2	92.9
**HQC**	1.1	100.4	1.1	93.8
**Interday (n = 3)**	**Precision (RSD%)**	**Accuracy (Er%)**	**Precision (RSD%)**	**Accuracy (Er%)**
**LQC**	1.8	100.9	0.6	99.3
**MQC**	0.2	96.4	2.0	98.7
**HQC**	0.8	94.7	0.6	97.3

LOD: 3.3 × (standard deviation of the response/slope of calibration curve); LOQ: 10 × (standard deviation of the response/slope of calibration curve); RSD, relative standard deviation (SD × 100/Mean); Low (LQC), Medium (MQC) and High (HQC) Quality Controls.
